# Establishment and application of a competitive enzyme-linked immunosorbent assay differentiating PCV2 antibodies from mixture of PCV1/PCV2 antibodies in pig sera

**DOI:** 10.1186/s12917-016-0802-9

**Published:** 2016-08-26

**Authors:** Shuizhong Han, Yan Xiao, Dingding Zheng, Yanli Gu, Yajie Xuan, Yudan Jin, Wenqiang Pang, Yuxin Huang, Xiangdong Li, Junhua Deng, Kegong Tian

**Affiliations:** 1 National Research Center for Veterinary Medicine, Luoyang, People’s Republic of China; 2 College of Animal Science and Veterinary Medicine, Henan Agricultural University, Zhengzhou, People’s Republic of China

**Keywords:** Porcine circovirus 2(PCV2), Recombinant cap protein, Virus-like particle, Competitive ELISA

## Abstract

**Background:**

Porcine cirovirus type 1 (PCV1) and type 2 (PCV2) are circulating in Chinese pig herds and the infected pigs develop antibodies to both viruses. Current commercial available ELISA kits cannot differentiate PCV2-specific antibodies from the mixtures of PCV1 and PCV2 antibodies in PCV1/2-infected or PCV2-vaccinated pigs. Therefore, the need for developing PCV2-specific ELISA methods is urgent to evaluate PCV2 antibody level in exclusion of PCV1 antibody interference after PCV2 vaccination.

**Results:**

Virus-like particles (VLPs) of PCV2 based on the recombinant Cap protein were expressed in *Escherichia coli*. A competing ELISA was established by using the VLPs as coating antigen and a PCV2-specific monoclonal antibody as the competing antibody. The competing ELISA was compared with the results obtained by using an immunoperoxidase monolayer assay on 160 serum samples. The sensitivity and specificity of this competing ELISA were determined as 96.5 and 96.0 %, at 2 standard deviation from the mean or 91.8 and 100 % at 3 standard deviations from the mean. Next, a serological survey of 1297 vaccinated serum samples collected from commercial pig herds in Beijing, Hunan and Henan provinces in China was conducted. The results showed that 85.9 % of sera having positive PCV2 antibodies.

**Conclusions:**

The competing ELISA we developed in this study was both sensitive and specific to PCV2 and was suitable for large-scale PCV2 antibody monitoring in exclusion of PCV1 antibody interference after PCV2 vaccination.

## Background

Porcine circovirus (PCV) was first identified as a noncytopathic contaminant of PK-15 cell, and subsequently classified in the family *Circoviridae* [1]. PCV is a spherical non-enveloped virus with a diameter of approximately 17 nm, and a single-stranded closed circular genomic DNA 1.7 kb in size [2]. There are two genotypes of PCV, namely PCV1 and PCV2. Serological analysis reveals that cross-reactivity exists between PCV1 and PCV2 [3]. PCV1 is widely known to be nonpathogenic agent, and no discernible pathogenic have been associated with PCV1 infection in swine [4]. Conversely, PCV2 is related to several diseases, such as postweaning multisystemic wasting syndrome (PMWS), porcine dermatitis and nephropathy syndrome (PDNS), porcine respiratory disease complex (PRDC), reproductive disorders, enteritis, and proliferative and necrotizing pneumonia (PNP), totally as porcine circovirus disease (PCVD) [5].

PCV2 genome contains two major open reading frames (ORFs): ORF1 and ORF2. ORF1 encodes the protein which involves in viral DNA replication, whereas ORF2 encodes an approximately 30 kDa immunogenic capsid (Cap) protein [6]. It was reported that the recombinant Cap protein could self-assemble to form virus-like particles expressed either in insect cells or *Escherichia coli* [6, 7]. The recombinant Cap protein reacted strongly with serum from PCV2-infected or PCV2-vaccinated pigs, which suggested that it was a good candidate antigen for the development of diagnostic assays [8, 9].

In order to detect PCV2 antibody in serum, the most common diagnostic methods include indirect fluorescent assay (IFA) and immunoperoxidase monolayer assay (IPMA) [10, 11]. However, these tests are not PCV2-specific due to the fact of antigenic cross-reactivity between PCV2 and PCV1. Meanwhile, these techniques are not only time-consuming and labor-intensive, but also require experienced technicians to judge the result arbitrarily. Compared with the current available methods, Enzyme-linked immunosorbent assay (ELISA) can be automated which decrease the potential bias and fit for mass detection. Several ELISA assays have been developed using the PCV2 virons or recombinant Cap protein expressed in insect cells [12–15].

In present study, a competitive ELISA (cELISA), using virus-like particles (VLP) of PCV2 rCap protein as the coating antigen and PCV2-specific monoclonal antibody (MAb) as the detecting antibody, was established. The establishment of this cELISA will facilitate to simply detect PCV2-specific antibodies from swine serum samples without PCV1 antibody interference.

## Methods

### PCV2 antigen and monoclonal antibody preparation

VLPs formed by recombinant Cap protein were produced in *E.coli* BL21 (DE3) strain as previously described [16] and used as the coating antigen for cELISA. Briefly, the supernatant of cell lysates containing recombinant Cap (rCap) protein was precipitated by 60 % saturated ammonium sulfate and resuspended, followed by anion ion-exchange chromatographic purification. The purified recombinant PCV2 Cap proteins have been completely re-assembled into VLPs in a buffer of 50 mM Tris–HCl and 500 mM NaCl. 200 μl (0.4 μg/μl) recombinant PCV2 Cap protein plus equal volume of Freund’s complete adjuvant was used as an immunogen to inject each of five female Balb/c mice (purchased from Vital Rivea Experimental Animal Technology Ltd., Beijing) via intraperitoneal injection for Mab production. Three booster immunizations with same dose of antigen plus Freund’s incomplete adjuvant were conducted at two-week intervals. Three days after the final booster injection, the mice were euthanized and spleen cells were fused with SP2/0 cells using standard procedure [17]. The hybridoma cells were maintained in RPMI1640 medium (Gibco, USA) with 17 % fetal bovine serum (Hyclone, USA). The supernatant of the hybridoma cells were harvested and tested for antibodies to PCV2 and PCV1 by IPMA. The colony of 3H11 MAb reactive to PCV2 but not to PCV1 tested by IPMA was subcloned two times and selected for use in the cELISA. The MAbs were labeled with horseradish peroxidase (HRP) according to the conventional methods [18].

### Serum samples

Five colostrum-deprived specific-pathogen-free piglets (Purchased from SPF Swine Breeding and Management Centre, Beijing) were intranasally inoculated with 10^5.0^ TCID_50_ infective doses of PCV2 SH strain. Serum samples were collected 0, 7, 14, 21, 28, 35, and 42 days post-vaccination (dpi) and separated for serological testing by IPMA and cELISA. Serum samples collected at 0 dpi worked as negative controls. One hundred and sixty clinical serum samples stored at National Research Center for Veterinary Medicine were tested by IPMA for cELISA development.

In the retrospective serologic study, a total of 1297 field pig serum samples were collected by Veterinary Diagnostic Laboratory from Beijing (377 samples), Hunan (432 samples) and Henan (488 samples) provinces in China. The experiments were carried out under the consent of animal owners. The serum samples were tested by the cELISA established in this study.

### Immunoperoxidase monolayer assay (IPMA)

IPMA was used to detect the presence of antibodies to PCV2. Briefly, the confluent monolayer of PKK cells (a PK-15 deprived cell line) infected with PCV2 SH (MOI=0.01) or free of PCV, were fixed in 80 % acetone for 30 min at 4 °C. The plates were stored at −20 °C after three times washing with PBS (0.01 mol/L, pH7.2). The MAb or serum samples were diluted 1:50 with PBS (0.01 mol/L, pH7.2), and added into the PCV2- and mock-infected PKK cells, respectively, 50 μl/well, and then incubated at 37 °C for 40 min. The anti-PCV polyclonal antiserum (VMRD, USA) and negative serum gained from colostrums-deprived piglets were respectively prepared as the positive and negative control. After three times washing, 50 μl of 1:200 dilution of HRP-conjugated goat anti-mouse or goat-pig IgG (Sigma, USA) was added and incubated at 37 °C for 30 min. After three times washing, 50 μl of the substrates 3-amino-9-ethylcarbazole was added and incubated for 30 min at room temperature. After three times washing, the 1:10 dilution hematoxylin was added. Twenty seconds later, the plates were washed with water and examined under an inverted light microscope.

### Development of cELISA

Optimized dilution of PCV2 VLP antigen and horseradish peroxidase-conjugated PCV2-specific MAb were established by systematic checkerboard titrations. The polystyrene microliter ELISA plates were coated with 1.6 μg PCV2 VLP in phosphate buffer (0.02 mol/L, pH7.4) at 4 °C for 16 ~ 24 h. After three times washing, the plates were blocked with 200 μl of 20 % calf bovine serum in phosphate buffer (0.02 mol/L, pH7.4) for 2 h at 37 °C. After three times washing, 50 μl of the serum samples were added to each well, then 50 μl of 1:2000 dilution MAb 3H11 conjugated with HRP (Sigma, USA) were added to the wells except the blank well. The plates were incubated at 37 °C for 30 min. After five times washing, 100 μl of the substrate solution (0.2 mg/ml of TMB and 0.2 % H_2_O_2_ in 0.05 mol/L citrate buffer, pH4.6) was added and the colorimetric reaction was developed at 37 °C for 15 min. The reaction was stopped by adding 50 μl of 2 mol/L sulphuric acid. The optical density (OD) was measured at 450 nm. The controls included positive control (in triplicate), negative control (in triple) and one blank control. The OD_450_ of the samples were converted to a percent inhibition (PI) value using the following formulation: PI (%) = (OD_450_ value of negative value − OD_450_ value of sample)/OD_450_ value of negative value × 100 %.

### Reproducibility, thermal stability and specificity of the cELISA

Inter-assay and intra-assay repeatability for the established cELISA was evaluated by testing the sixty filed sera. For the inter-assay repeatability, three replicates of each serum samples were detected by the same batch of pre-coated ELISA plates. For the intra-assay repeatability, each serum samples were detected by three batches of pre-coated ELISA plates. Mean PI value and coefficient of variation (CV) of three replications of each test were calculated.

Thermal stability for the established cELISA was evaluated by testing five filed serums using the plates stored at 4 and 37 °C for six days, respectively. The PI value of each serum detected with the plates stored at 37 °C for six days was compared with those had been stored at 4 °C. Statistical analysis of the PI value was carried out by Student^’^s test using the SPSS 19.0 software. The significance level was set at 0.05 (*p < 0.05*).

To explore the specificity, positive sera for PCV1, classical swine fever virus (CSFV), high pathogenic porcine reproductive and respiratory syndrome virus (HP-PRRSV), porcine parvovirus (PPV), and porcine pseudorabies virus (PRV) were tested with the established cELISA.

## Results

### Sera from experimentally infected piglets

Standard PCV2-negative and positive serum samples were house-made by infecting pig with PCV2. Sera from these pigs sampled at 28, 35 and 42 day post-infection (dpi) were reactive with PCV2-infected cells by IPMA and were used as PCV2-postive serum samples. Sera at 0, 7, and 14 dpi were not reactive with PCV2 and were used as negative serum samples in this study. The positive sera had OD_450_ values < 0.719 when tested by cELISA established in this study with the inhibition in excess of 77.8 %. By contrast, the negative sera had OD_450_ values larger than 1.270 with inhibition less than 60.8 %.

### Determination of the cut-off of cELISA by using clinical sera

**Fig. 1:**
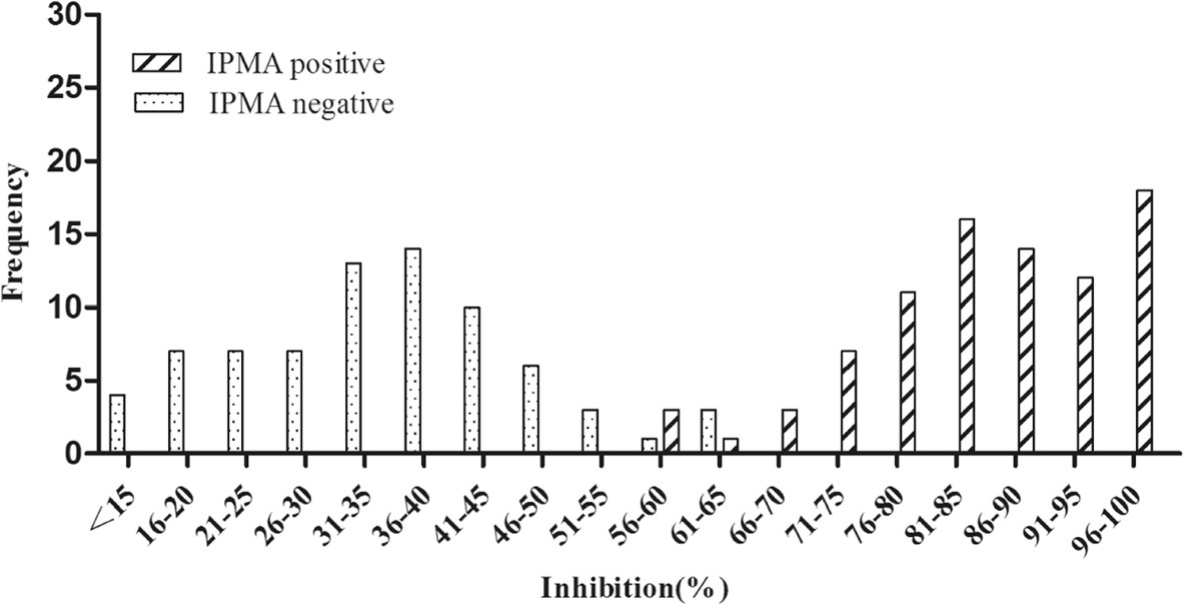
Distribution of the cELISA PI values for detection of IPMA positive and negative pig sera. The horizontal bar shows the frequency of IPMA-positive and -negative samples and the vertical bar shows the inhibition values

A total of 160 field serum samples tested by IPMA were used to determine the cut-off value of established cELISA (Table 1). Among them, 75 samples were confirmed to be serological PCV2-negative by IPMA. These serum samples were tested by the established cELISA in this study. Distributions of the cELISA PI values showing the frequency of IPMA-positive and -negative samples are shown in Fig. 1. The average PI values (x-axis) of the 75 IPMA negative sera detected by cELISA were 34.4 % ± 12.0 % (mean ± SD). When 58.4 % (mean + 2SD) was used as the cutoff value for percentage inhibition, the sensitivity was 96.5 % and specificity was 96.0 %, giving a 95 % confidence. When the cutoff increased to 70.4 % (mean + 3SD), the sensitivity and specificity of the cELISA was 91.8 and 100 %, giving a 99 % confidence.

**Table 1:** Determination of sensitivity and specificity of the cELISA using cut-off values of the mean negative PI plus two SD or three SD

cELISA*	IPMA*		
	Positive	Negative	Total
Positive	82^a^	3^a^	85^a^
	78^b^	0^b^	78^b^
Negative	3^a^	72^a^	75^a^
	7^b^	75^b^	80^b^
Total	85	75	160

*Note*:*IPMA immunoperoxidase monolayer assay, *cELISA* competitive enzyme-linked immunosorbent assay

^a^Comepetitive ELISA positive 58.4 % inhibition or greater

^b^Comepetitive ELISA positive 70.4 % inhibition or greater

### Reproducibility, thermal stability and specificity of the cELISA

The reproducibility of the cELISA was determined by calculating the coefficient of variation (CV) of the PI values from field serum samples. The inter-assay CV of the 40 PCV2-positive serum samples ranged from 0.06 to 4.62 %, while the intra-assay CV of those same samples ranged from 0.16 to 7.46 % (Table 2). The inter-assay and intra-assay of the 20 negative samples also exhibited good repeatability, showing 2.2–14.8 % and 5.1–14.6 % respectively. These data showed that this assay was repeatable with low and acceptable variations.

**Table 2:** Coefficient values of the 60 field sera tested by the cELISA

Inhibition range(%)	No. of sera tested	CV range (%)	
		Inter-assay	Intra-assay
81–100	20	0.06–2.31	0.16–7.46
70–80	20	0.8–4.62	0.64–5.31
<60	20	2.2–14.8	5.1–14.6

The thermal stability assay was performed by comparing the PI values of 5 PCV2 positive serum samples on different ELISA plates which have been stored at 4 and 37 °C for six days. The results showed there was no significant difference (*p* = 0.107) existed between these two temperature conditions.

In addition, the cross-reactivity of the cELISA with several positive sera against CSFV, HP-PRRSV, PPV, PRV and PCV1 was also tested according to the cELISA procedures. The OD_450_ values of all these sera were in excess of 1.930 (PI ≤ 38.5 %) and were negative.

### Serological survey of PCV2-vaccinated pigs using the established cELISA

The results for the 1297 field serum samples, collected from three provinces in China are shown in Table 3. Of these, 1106 samples were positive (85.3 %) and 191 samples (14.7 %) were negative for PCV2 antibodies. Regionally, the positive rate was 80.6 % (348/432) in Hunan province, 79.3 % (387/488) in Henan province and 98.4 % (371/377) in Beijing city.

**Table 3:** Prevalence of antibodies to PCV2 in pig sera collected from Henan, Hunan, and Beijing

Areas	Positive	Negative	Total	Positive rate
Beijing city	371	6	377	98.4 %
Hunan province	348	84	432	80.6 %
Henan province	387	101	488	79.3 %
Total	1106	191	1297	85.3 %

## Discussion

PCV2 is currently circulating worldwide and leads to huge economic losses to swine industries. The commonly used and most effective strategy to control this disease is vaccination. The antibodies elicited by vaccination are mainly targeted to the PCV2 Cap protein, the sole and highly immunogenic structural protein of virus. Therefore, evaluation of PCV2 antibodies after virus infection or vaccination may reflect the herd infection status or the efficacy of vaccines. Currently, the common analytical methods developed for the qualitative and quantitative analysis of PCV2 antibodies in serum samples are IIF and IPMA. However, the antibodies detected by IIF and IPMA were not PCV2-specific due to the antigenic cross-reactivity between the Rep proteins encoded by PCV2 and PCV1 [19]. Therefore, development of specific and convenient methods for PCV2 antibody evaluation is more meaningful in PCV2 serological test.

It was previously reported that ELISA coated with the recombinant PCV2 Cap protein or peptide could be used for the specific detection of PCV2 antibody [8, 20]. Several indirect ELISA methods using the recombinant Cap protein have been developed for detecting the presence of PCV2 antibody in pig sera [12–14]. However, the indirect ELISA has some limitations including the high level of false positives due to the complex components of serum samples such as Rheumatoid factor and antibodies specific for other animal serum components. Compared with indirect ELISA, cELISA have distinct advantages. First, the secondary antibody against the tested species is nonessential. Secondly, the nonspecific background caused by the tested sera could be lower by using the specific anti-PCV2 MAb. A cELISA have been previously established for the detection of PCV2 antibodies [15]. In their studies, live PCV2 virions from the cell cultures have been used as the coating antigen. The use of live virus has the issue of biosecurity and the production of live virus as antigen is laborious and time-consuming. Therefore, in our study, *E. coli*-originated PCV2 VLPs have been used as coating antigen with the advantages of intact antigenicity and easier preparation.

Besides the PCV2 VLPs used as coating antigen in this study, a PCV2-specific MAb was employed as a competitor with test sera. The MAb used in this assay is not reactive with PCV1 which exclude the inference of PCV1 antibodies that existed in the serum samples. Both the serum samples from experimental infected with PCV2 and from the clinical animals were used to establish the cELISA. The sensitivity and specificity of the cELISA was determined by detecting 160 field serums which have been confirmed IPMA. When the cutoff value of this cELISA was set to 58.4 % inhibition, three IPMA positive/cELISA negative serum samples were detected. These serum samples may contain antibodies to different stain of PCV2 that were reactive by IPMA but not with cELISA since a wide range of viral epitopes could be recognized by the polyclonal antibodies in IPMA. Three IPMA negative/cELISA positive samples were also detected. This discrepancy may be explained by the higher sensitivity of cELISA than IPMA. When the cutoff increased to 70.4 % inhibition, the IPMA negative/cELISA positive disappeared and the numbers of IPMA positive/cELISA negative serums increase to seven. Hence, the gray zone of the cELISA was set to be in the region of 58.4 to 70.4 %, and the samples should be retested when PI values fall into the range.

The established cELISA was next applied to do the PCV2 serological survey by using 1297 serum samples collected from three different provinces. The results of the survey reveals the PCV2 antibody positive rates ranged from 79.3 to 98.4 % which indicates the good herd immunity after vaccination.

## Conclusions

To summarize, a cELISA was successfully established in this study to specifically detect PCV2 antibodies in exclusion of PCV1 antibody interference in serum samples. The specificity and convenience of this cELISA will be a useful tool to evaluate the strength of post-vaccination antibody response and monitor the efficacy of current vaccines.

## Abbreviations

PCV2: Porcine cirovirus 2
PCV1: Porcine cirovirus 1
ORFs: Open reading frames
ELISA: Enzyme-linked immunosorbent assay
Cap: Capsid
IFA: Indirect fluorescent assay
IPMA: Immunoperoxidase monolayer assay
cELISA: Competitive enzyme-linked immunosorbent assay
VLP: Virus-like particles
MAb: Monoclonal antibody
*E.coli*: *Escherichia coli*
HRP: Horseradish peroxidase
TCID_50_: Tissue culture infective dose
Dpi: Days post-vaccination
PBS: Phosphate-buffered saline
OD: Optical density
PI: percent inhibition
iELISA: Indirect enzyme-linked immunosorbent assay
